# Uncovering molecular events associated with the chemosuppressive effects of flaxseed: a microarray analysis of the laying hen model of ovarian cancer

**DOI:** 10.1186/1471-2164-15-709

**Published:** 2014-08-24

**Authors:** Karen H Hales, Sheree C Speckman, Nawneet K Kurrey, Dale B Hales

**Affiliations:** Department of Obstetrics and Gynecology, Southern Illinois University at Carbondale, School of Medicine, Life Science III, (M/C 6512), 1135 Dr., Carbondale, Lincoln, IL 62901 USA; Department of Physiology, Southern Illinois University at Carbondale, School of Medicine, Life Science III, (M/C 6512), 1135 Dr., Carbondale, Lincoln, IL 62901 USA; CSIR Central Food Technological Research Institute, Mysore, KA 570020 India

**Keywords:** Ovarian cancer, Laying hen, Flaxseed, Branching morphogenesis

## Abstract

**Background:**

The laying hen model of spontaneous epithelial ovarian cancer (EOC) is unique in that it is the only model that enables observations of early events in disease progression and is therefore also uniquely suited for chemoprevention trials. Previous studies on the effect of dietary flaxseed in laying hens have revealed the potential for both amelioration and prevention of ovarian cancer. The objective of this study was to assess the effect of flaxseed on genes and pathways that are dysregulated in tumors. We have used a bioinformatics approach to identify these genes, followed by qPCR validation, immunohistochemical localization, and in situ hybridization to visualize expression in normal ovaries and tumors from animals fed a control diet or a diet containing 10% flaxseed.

**Results:**

Bioinformatic analysis of ovarian tumors in hens led to the identification of a group of highly up-regulated genes that are involved in the embryonic process of branching morphogenesis. Expression of these genes coincides with expression of E-cadherin in the tumor epithelium. Levels of expression of these genes in tumors from flax-fed animals are reduced 40-60%. E-cadherin and miR200 are both up-regulated in tumors from control-fed hens, whereas their expression is decreased 60-75% in tumors from flax-fed hens. This does not appear to be due to an increase in ZEB1 as mRNA levels are increased five-fold in tumors, with no significant difference between control-fed and flax-fed hens.

**Conclusions:**

We suggest that nutritional intervention with flaxseed targets the pathways regulating branching morphogenesis and thereby alters the progression of ovarian cancer.

**Electronic supplementary material:**

The online version of this article (doi:10.1186/1471-2164-15-709) contains supplementary material, which is available to authorized users.

## Background

One out of 71 women will develop ovarian cancer in her lifetime. The five year survival rate is less than 44%, making ovarian cancer the most lethal gynecologic malignancy. This number has not changed significantly in the last 20 years in spite of advances in platinum-based chemotherapy [1]. For this reason, there is a critical need to explore effective chemoprevention strategies.

It has been estimated that at least 30% of all cancers could be prevented through diet, exercise, and maintaining a healthy weight [2]. One outcome of this approach is the reduction of the chronic, low-grade systemic inflammation that accompanies obesity. Chronic inflammation has been implicated to play a causative role in many diseases, including cancer [3]. Further reduction of inflammation can be achieved by lowering the ratio of omega-6 to omega-3 fatty acids. The modern western diet contains a high ratio of omega-6 to omega-3 fatty acids, a profile that is both pro-inflammatory and oxidant-rich and creates an environment conducive to the development of disease. Flaxseed is one of the richest plant sources of omega-3 fatty acids. In addition, flaxseed also contains lignans, a class of phytoestrogens that also act as antioxidants [4]. These two different nutriceuticals have pathway-specific actions, targeting inflammation and oxidative damage.

Research into the etiology of ovarian cancer has been limited by the lack of suitable animal models. The laying hen is a robust model in that ovarian cancer develops spontaneously with pathological and histological presentation very similar to human disease [5, 6]. As in women, the average age of onset occurs later in reproductive life, with 40% of hens having the disease by six years of age [7]. The disease can progress rapidly, with transcoelomic spread disseminating from the ovary to organs and peritoneal surfaces, and with the accumulation of ascites. The four histotypes observed in human are represented in the hen, although the endometrioid type is the predominant form found in the hen whereas the serous type is most prevalent in women [8]. Mutations in p53 are common in epithelial ovarian cancer (EOC) from both species [9]. Numerous characteristic markers are also shared between the tumors of the two species such as CA-125 [10], CYP1B1 [11], E-cadherin [12] and COX-1 [13]. The expression of COX-1 and accompanying high levels of prostaglandin E2 presents a target for dietary intervention with omega-3 fatty acids. Our one year study of hens fed a diet of 10% flaxseed showed reduction in cancer severity that corresponded to a reduction in prostaglandin levels [14]. This suggests that ovarian cancer progression may be driven by inflammation. Our long term study in which hens were fed a diet supplemented with 10% flaxseed for four years resulted in a significant decrease in both incidence and severity of ovarian cancer [15]. This suggests that in addition to decreased progression, initiation and/or promotion of this disease may be slowed by some component of flaxseed. This data is dually important in that it highlights the utility of the hen model for use in dietary studies of chemoprevention, and it provides strong evidence that dietary flaxseed significantly affects the initiation, promotion and progression of ovarian cancer. Thus, identification of the pathways altered by flaxseed may give insight into the etiology of the disease.

The objective of the current study was to identify possible targets and pathways affected by dietary flaxseed and by ovarian cancer to determine the mechanisms by which flaxseed confers chemoprotection against ovarian cancer. We performed a microarray analysis which compared normal ovaries and ovarian tumors from hens fed a control diet to those of hens fed a diet supplemented with 10% flaxseed. Microarray analysis was followed by comprehensive bioinformatics and several levels of experimental validation. This analysis revealed that pathways associated with branching morphogenesis are significantly increased in ovarian cancer and reduced by flaxseed, suggesting that the process driving tumor growth and progression toward a glandular morphology are targeted by the biologically active constituents of flaxseed.

## Results and discussion

### Flaxseed modulates genes linked to ovarian cancer in the laying hen

**Figure 1:**
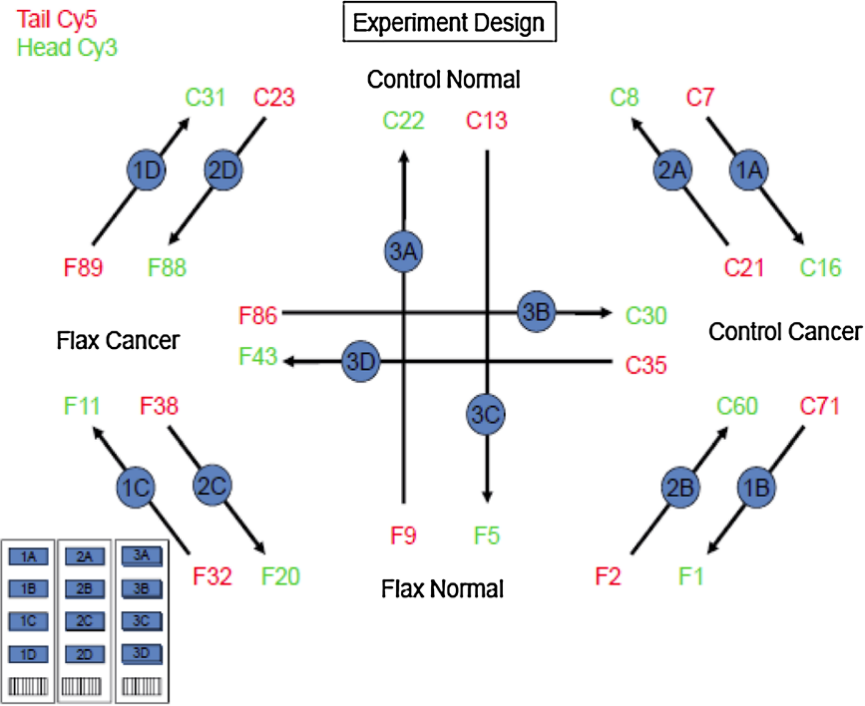
**Diagrammatic representation of microarray experimental design.** Based on 2X2 experimental conditions i.e. diet (control & Flaxseed enriched) and tissue (normal & cancer) 44 k arrays were designed. The arrow represents Cy3; the end of the arrow represents Cy5.

**Figure 2:**
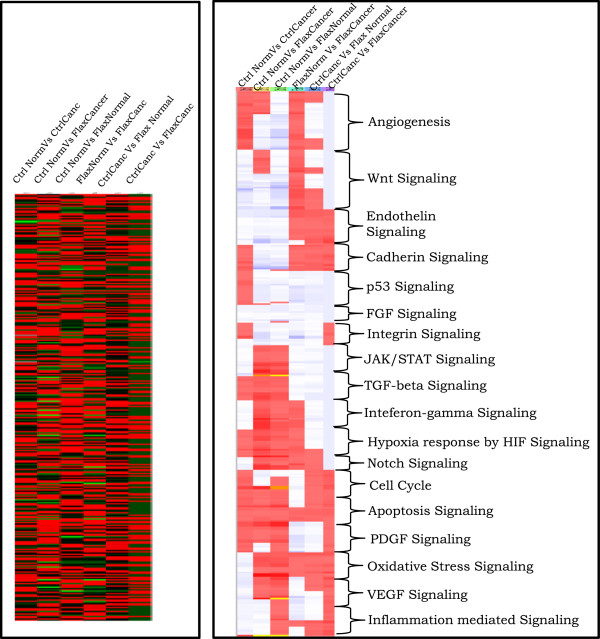
**Microarray analysis showing the effects of flaxseed in ovarian cancer at whole genome level.** Left, chicken whole genome wide heat map expression pattern at global level: All the gene probes used in the array which showed detectable level of expression during analysis were visualized using heat map to show their differential expression levels between the groups i.e. control-cancer vs. control-normal, control-cancer vs. flax-normal, control-cancer vs. flax-cancer, flax-cancer vs. flax-normal, flax-cancer vs. control normal and flax-normal vs. control normal. Right, heat map visualization of pathway associated genes: Genes representing the specific biological pathway involved in cancer progression were sorted and their expression levels were examined between the different analysis group and a heat map was generated for visualization.

To evaluate the genome-wide effect of flaxseed in ovarian cancer, we conducted microarray and bioinformatics analyses. The Agilent custom 4x44K chicken long oligo microarray [16] was utilized for this study based on 2X2 experimental conditions, i.e. diet (control & flaxseed enriched) and tissue (normal & cancer). The samples were obtained from the one year flaxseed study [14]. We performed a 6-way pair analysis of the gene list between the following groups: 1. Control Cancer (CC) to Control Normal (CN); 2. Control Cancer to Flax Normal (FN); 3. Flax Normal to Flax Cancer (FC); 4. Flax Cancer to Control Normal; 5. Control Normal to Flax Normal; 6. Flax Cancer to Control Cancer (Figure 1). The gene expression pattern in each analysis group is distinct and separates one group expression pattern from another, suggesting the effect of flaxseed is diverse at the gene level (Figure 2). This result encouraged us to focus on specific sets of genes which are linked to cancer progression. Annotated genes used in the 4X44K arrays were classified based on their biological and molecular pathways using a pathway analysis tool which yielded 337 genes (list of genes in Additional file 1) related to the pathogenesis of ovarian cancer [17]. Further, we measured the expression of these 337 genes in the CC, CN, FN, & FC groups and pair wise analyses were carried out between the groups (Figure 2). Comparing the expression in different sample groups suggests that the group fed with flaxseed downregulated cancer promoting pathways such as angiogenesis, VEGF signaling, endothelin signaling, WNT signaling, cadherin signaling, inflammation and oxidative stress signaling. Similarly, tumor suppressor pathways such as p53 signaling, apoptosis signaling, cell cycle, and JAK/STAT signaling were upregulated, indicating that the flaxseed acts as an overall tumor suppressor by negatively regulating the ovarian cancer associated pathways and promoting tumor suppressor pathways.

### Microarray identification of genes responsive to flaxseed

**Figure 3:**
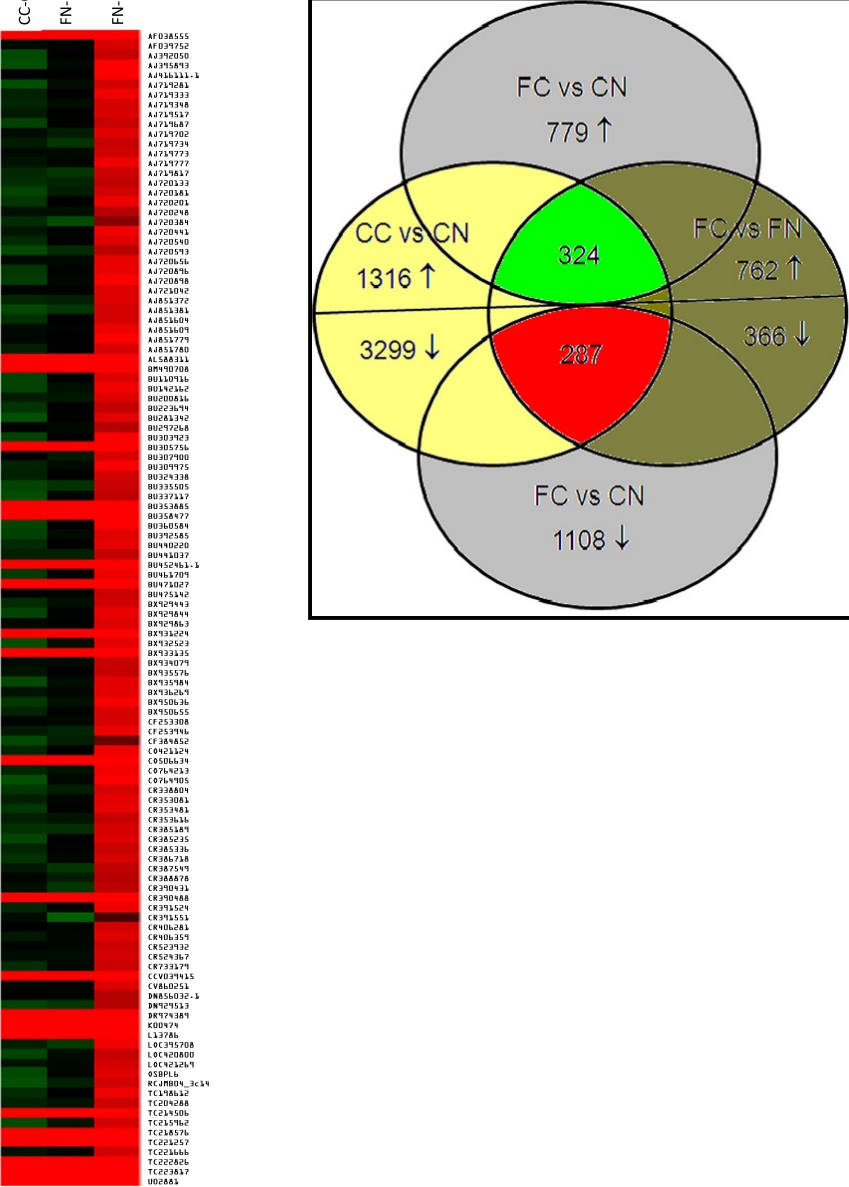
**Analysis of genes affected by cancer and diet.** Left, changes in the expression of sorted genes between flax-normal vs. control-cancer (FN-CC) was visualized by heat map. Right, Venn diagram of overlapping genes among the different data sets viz. control-cancer vs. control-normal (CC-CN), flax-cancer vs. flax-normal (FC-FN) and flax-normal vs. control-normal (FN-CN) dataset. Genes are listed in additional file 2.

In order to identify the genes which may be directly linked with ovarian cancer progression, a comparison was made of all cancer groups (control cancer and flax cancer) to all normal groups (control normal and flax normal) i.e. CC-CN to FC-FN, CC-CN to FC-CN and FC-FN to CC-FN. This exercise generated 324 upregulated genes and 287 downregulated genes which were common in both cancer groups (control cancer and flax cancer), indicating that their differential expression may be involved in ovarian cancer pathogenesis (Figure 3). In order to examine the flaxseed responsive genes, we filtered and sorted a list of 118 genes (see material & methods) from our microarray analysis which are involved in ovarian cancer and may be targets of flaxseed. Further examination of the expression levels of these 118 genes in CC-CN and FN-CN (Additional file 2) revealed that there is differential expression between the groups. The genes exhibit a difference in magnitude of expression (fold change), indicating that the degree of difference in expression is crucial for cancer progression (Figure 3). Genes that are upregulated in cancer are also increased in tumors from flax-fed animals but not to the same extent. This is in agreement with earlier observations demonstrating that the expression of certain genes above a critical threshold plays a protective role against carcinogenesis but their aberrant expression beyond this critical level may have an adverse effect [18]. Comparison of 118 genes between flax-normal vs. control-cancer showed that most of these genes have significant differences in their expression levels.

### Real time PCR validation corroborates flaxseed responsive genes found by microarray analysis

**Figure 4:**
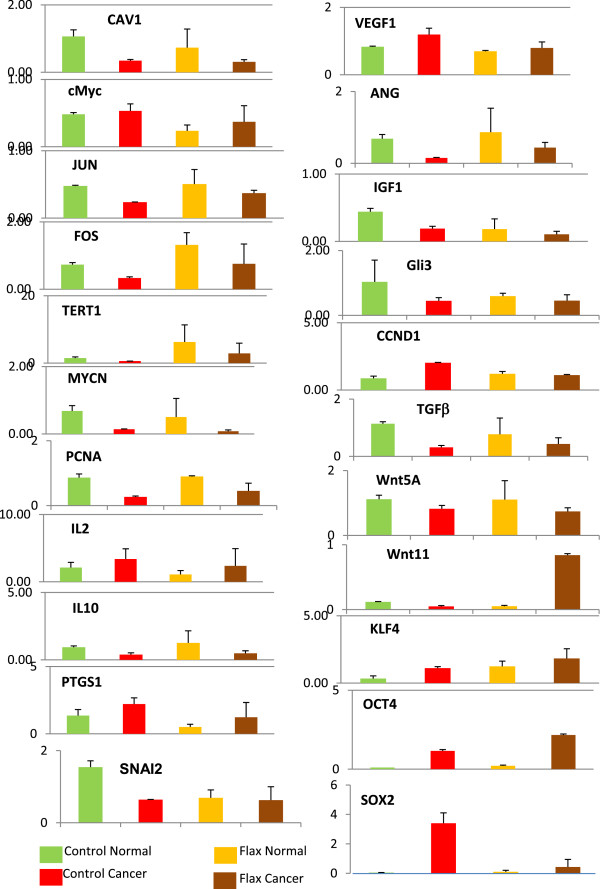
**Real Time-PCR gene expression analysis.** Selected genes were analyzed by PCR-array to validate microarray data.

To validate our microarray analysis, we evaluated the mRNA levels of 44 genes by PCR-array. These were representative genes identified by pathway analysis to be involved in angiogenesis, signaling (wnt, integrin, cadherin, JAK/STAT, Notch, VEGF pathways), cell cycle, apoptosis, inflammation, oxidative stress and developmental pathways (Table 1). Figure 4 shows the expression of 22 genes in each sample group. The level of gene expression of these genes quantified by qPCR significantly matches with the array results (not shown). Of the 22 genes not shown, four are the house keeping genes used for normalization, five were selected for further validation, and 13 were not significantly different between groups. One of the important observations of the study was that the well-established cancer promoting and inflammatory genes like *IL2, VEGF1, IGF1, PTGS1, cMYC* and *CCDN1* [19] were modulated by flaxseed compared to control-fed hens. Reduction in the expression of these inflammatory genes suggests that flaxseed mitigates cancer promoting activities and acts as an anticancer agent. Stemness-associated genes *KLF4, OCT4* and *SOX2* [20] were also analyzed. *SOX2*, *KLF4* and *OCT4* were elevated in tumors from control-fed hens. However, in tumors from flax-fed hens, only *SOX2* was greatly reduced whereas both *KLF4* and *OCT4* expression was enhanced. Thus, flaxseed did not have a uniform effect on the expression of genes associated with stem cells.

**Table 1:** **Genes included in PCR array**

Gene	Function
Jun	Fos and Jun dimerize to form AP-1, involved in cell proliferation, differentiation, and transformation
Fos	
PCNA, proliferating cell nuclear antigen	DNA synthesis, cell-cycle control, and DNA-damage response and repair
CCND1, cyclin D1	Regulatory subunit of CDK4 or CDK6, whose activity is required for cell cycle G1/S transition
TERT1, telomerase reverse transcriptase	Maintains telomere ends, chromosomal repair
mycN	Transcription factor amplified or overexpressed in variety of tumors
cMYC	Transcription factor activated upon various mitogenic signals such as Wnt, Shh and EGF
gli3	Transcription factor mediating sonic hedgehog signaling
SOX2	Transcription factor with roles in embryonic development, cell fate determination, stem cell maintenance
Oct4	Involved in the self-renewal of undifferentiated embryonic stem cells.
Klf4	Indicator of stem-like capacity in embryonic stem cells
VEGF1	Endothelial cell mitogen
ANGPT1, angiopoietin 1	Involved in vascular development and angiogenesis
CAV1, caveolin	Scaffolding protein, possible tumor suppressor
Wnt5A	Secreted signaling protein, activates beta catenin transcriptional activity
Wnt11	Secreted signaling protein, implicated in oncogenesis and in several developmental processes
SNAI2, snail2	Transcriptional repressor involved in epithelial-mesenchymal transitions and has antiapoptotic activity.
TGFbeta	Member of a family of peptides that regulate proliferation, differentiation, adhesion, migration, and other functions in many cell types
IGF1, insulin like growth factor	Growth and anabolic effects
IL2	Cytokine regulates lymphocyte activity
IL10	Anti-inflammatory cytokine
PTGS1, COX1, prostaglandin G/H synthase and cyclooxygenase)	Converts arachidonic acid to prostaglandin

### E-cadherin, *PAX2, MSX2, FOXA2*and *Engrailed-1*are upregulated in hen ovarian cancer and decreased by dietary flaxseed

**Figure 5:**
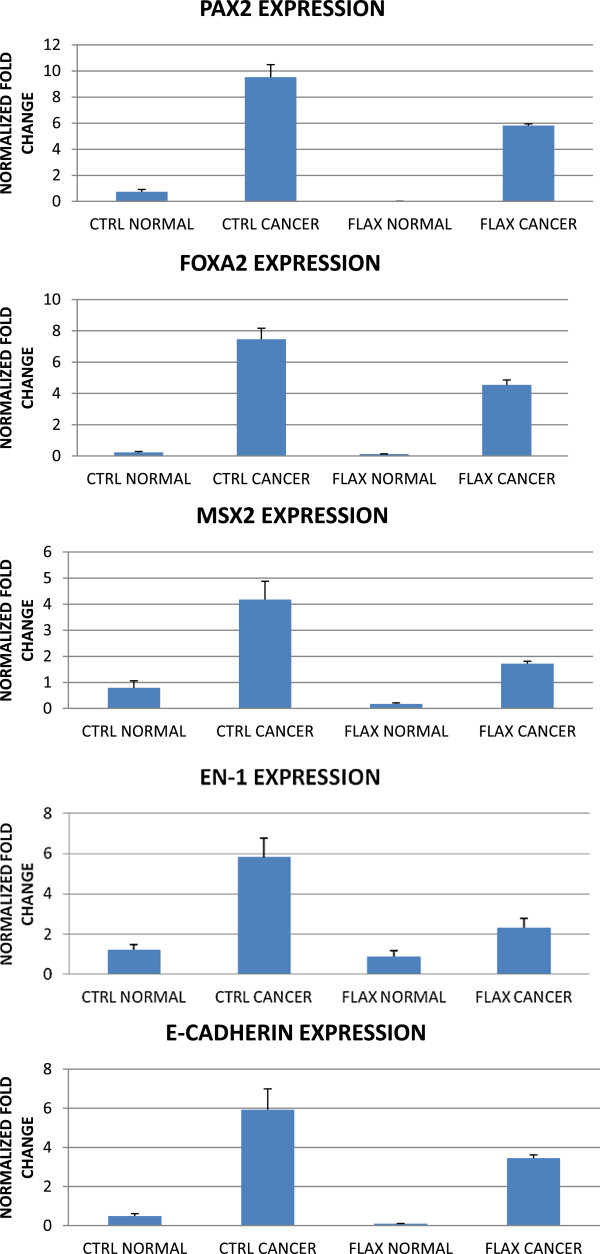
**PCR analysis of select genes shown to be differentially expressed by microarray.** *MSX2, FOXA2, EN-1, PAX2* and E-cadherin are significantly upregulated in ovarian cancer compared to normal ovaries. This upregulation is attenuated in ovarian tumors from flax-fed hens.

**Figure 6:**
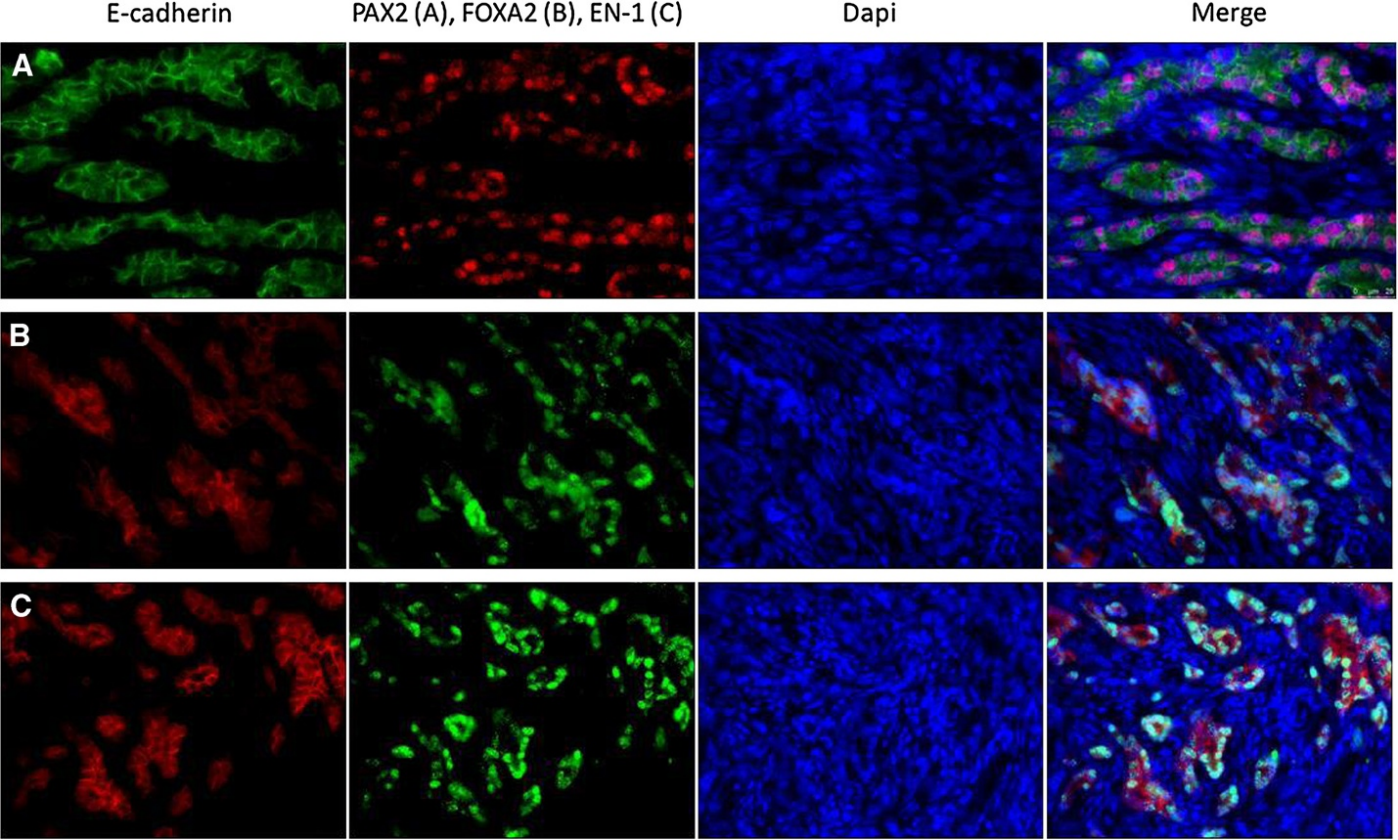
**Double fluorescent immunohistochemistry, Expression of** ***PAX2*** **(row A),** ***FOXA2*** **(row B) and** ***EN-1*** **(row C) colocalize with E-cadherin in glandular epithelial tumor cells.** Only E-cadherin is expressed in normal ovarian tissue; PAX2, FOXA2 and EN-1 are not (data not shown). Dietary flaxseed does not alter the spatial expression of these factors (data not shown).

The gene that showed the greatest induction by the microarray analysis was E-cadherin, with a 44-fold increase in tumors compared to normal ovary. Up-regulation of E-cadherin expression is one of the earliest events in the development of EOC and is common to all ovarian cancer histotypes [21]. This E-cadherin pattern could potentially have diagnostic value, but more importantly, identifying factors that regulate E-cadherin expression in the ovary may give clues as to which pathways become dysregulated very early on during the transformation of normal cells toward a malignant phenotype. We have shown previously that E-cadherin is significantly upregulated in hen ovarian cancer similar to what is observed in the human disease [12]. qPCR analysis from the current study reveals that E-cadherin mRNA is decreased more than 40% in tumors from hens fed flaxseed (Figure 5) and E-cadherin protein has been shown to be decreased by 50% in tumors from flax-fed hens [22]. E-cadherin is expressed in the ovarian surface epithelium (OSE) and expression in the tumor compartment is confined to the glandular epithelial cells (Figure 6). The observation that flaxseed decreases E-cadherin in ovarian cancer reveals that flaxseed modulates very early events in neoplastic transformation.

The microarray analysis and PCR array identified an additional group of genes that were upregulated in cancer and targeted by flaxseed. These were genes encoding transcription factors involved in early development, cell-fate determination and morphogenesis including *PAX2* [23] *, FOXA2* [24] *, MSX2* [25] and *EN1* [26]. The expression of these genes was assayed in samples obtained from the five year study (Figure 5) [15]. In agreement with data from both human [27] and chicken [28], we find *PAX2* expression is upregulated 9-fold in ovarian cancer from control-fed hens compared to normal ovaries from control-fed hens. Additionally, we show that flaxseed attenuated this upregulation in ovarian tumors to 6-fold compared to normal ovaries. *MSX2*, a member of the muscle segment homeobox family, is upregulated 5-fold in ovarian tumors from control-fed hens compared to normal ovaries. Dietary flaxseed significantly decreased this upregulation to 2-fold in ovarian tumors. *FOXA2* mRNA is aberrantly over-expressed 7-fold in ovarian tumors from control-fed hens. This upregulation is decreased by flaxseed to 4-fold in ovarian tumors compared to normal ovaries. Lastly, we show that tumors from control-fed hens exhibit a 6-fold upregulation of *EN1* mRNA compared to normal ovaries, and this upregulation is decreased by flaxseed to 2-fold compared to normal ovaries. In addition, the flaxseed diet has an inhibitory effect on the expression of *PAX2, MSX2* and E-cadherin mRNA in normal ovaries in the absence of pathology. That all of these genes are significantly downregulated in tumors from flax-fed hens suggests that they may play a role in the progression of the disease.

Immunohistochemical localization of *PAX2, FOXA2* and *EN1* reveals that they co-localize with E-cadherin in the glandular epithelial compartment of the ovarian tumor (Figure 6). No expression of these factors can be detected prior to the expression of E-cadherin in the cortical region of the ovary, nor do they co-localize with E-cadherin in the OSE. Three of these genes play an active role in normal gland morphogenesis. In the mouse, PAX2 protein is required for Mullerian duct formation, including ductal and mesenchymal elements [23]. *MSX2* expression has been reported to be increased in human ovarian endometrioid adenocarcinoma as a target of WNT signaling [29]. It has also been shown to play a role in branching morphogenesis during mouse mammary gland development through the action of BMP signaling [25]. It plays a role in both growth and apoptosis, particularly affecting the proliferative and regenerative capacity of tissue [30]. *FOXA2* plays a role in mouse lung morphogenesis [31] as well as chicken oviduct development [32], where it is modulated post-transcriptionally by estrogen. It promotes epithelialization during embryogenesis [33] and has been shown to directly [34] and indirectly [35] regulate E-cadherin expression. Engrailed has been best characterized in the Drosophila wing for its role as a segment polarity gene that transcriptionally activates Hedgehog, which in turn establishes the Decapentaplegic (BMP homolog) morphogen gradient [36]. In mammals, *EN2* has been shown to be dysregulated in bladder, ovarian and prostate cancers, whereas *EN1* has been demonstrated in salivary gland adenoid cystic carcinoma [37]. Interestingly, Engrailed can be secreted as well as taken up by cells and urinary EN2 levels have been proposed as a marker for prostate cancer [38]. Although expression of these genes may suggest a cell or tissue of origin for ovarian cancer, an alternative interpretation may be the induction of a morphogenic process in the ovary to which a plastic transformed cell responds. Thus, EOC could conceivably develop in the ovary due to the activation of morphogens responsible for glandular differentiation acting on transformed cells. The parallel patterns of expression we observe in these genes in tumors from both control-fed and flax-fed hens suggests that formation of EOC is mediated in part by aberrant activation of a developmental program which controls branching morphogenesis, and that dietary flaxseed impedes or perturbs this program.

### miR-200 family is upregulated in hen ovarian cancer compared to normal ovaries and is decreased by dietary flaxseed

**Figure 7:**
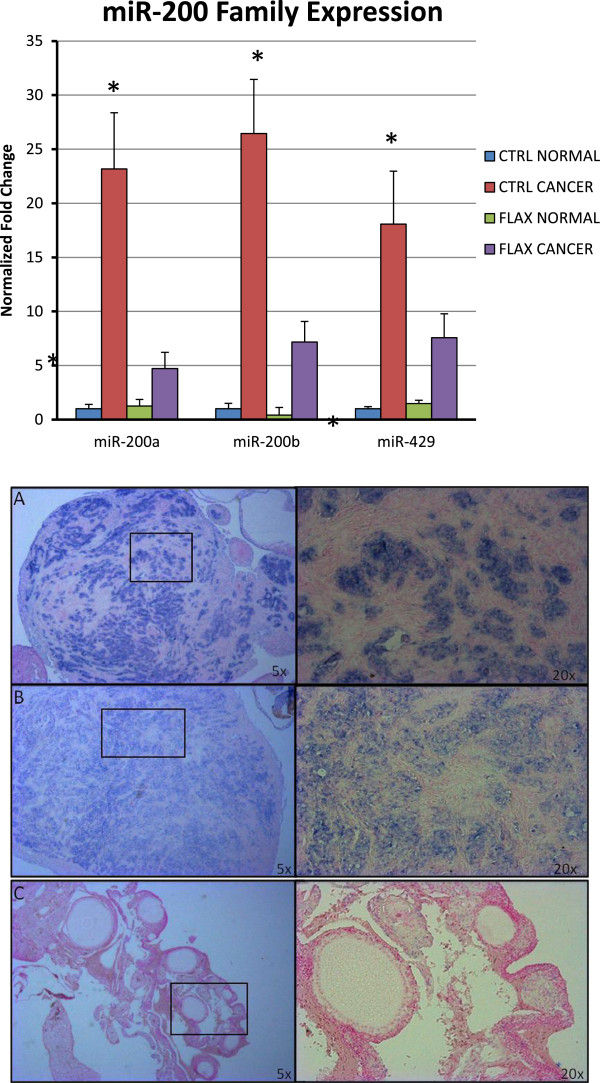
**qPCR analysis of miR-200 family and in situ hybridization of miR200a.** Expression of all three members of the chicken miR-200 family is significantly upregulated in ovarian tumors compared to normal ovaries. Expression of all three members is significantly decreased in ovarian tumors from flax-fed hens compared to tumors from control-fed hens. Expression of miR-200a is confined to the tumor cells of the control-fed ovary **(A)** and flax-fed ovary **(B)**. Little if any expression is observed in normal ovary tissue **(C)**. Magnification 20x on left, 100x on right.

It has been observed in human ovarian cancers that increased E-cadherin parallels increased levels of miR-200 family members [39]. While the function of the miR-200 family in ovarian cancer is complex, this upregulation is currently under rigorous scrutiny for potential diagnostic and prognostic value. To date, the relationship between E-cadherin and the three members of the chicken miR-200 family has not been examined in chicken ovarian cancer. We measured expression levels of miR-200a, miR-200b, and miR-429 in ovarian tumors from control-fed hens and found they were upregulated 22-fold, 26-fold, and 18-fold respectively, compared to expression levels in normal ovaries. Expression levels of miR-200a, miR-200b and miR-429 were upregulated 5-fold, 7-fold, and 8-fold respectively, in ovarian tumors from flax-fed hens compared to normal ovaries (Figure 7). These data show that the chicken miR200 family is upregulated in ovarian tumors and that flaxseed was able to significantly inhibit the up-regulation of all three members in ovarian cancer by 55 to 80%. It has recently been shown that miR200 family members can be induced in ovarian cancer cells after exposure to oxidative stress [40]. Flaxseed acts as an antioxidant, particularly through the action of the lignan seicoisolariceresinol diglucoside, and its metabolites enterodiol and enterolactone [41, 42]. The action of these antioxidants may account for the decreased levels of miR200 family members in the flax-fed hens. Localization of miR-200a by in situ hybridization shows that expression is confined to the glandular epithelial compartment of the ovarian tumor, reflecting the positive correlation between miR-200 expression and E-cadherin expression in hen ovarian cancer (Figure 7). Relatively few studies have shown that dietary manipulation can directly affect miRNA expression. A limited number of specific dietary constituents and phytochemicals have been identified which show direct or indirect chemopreventive or chemotherapeutic action by modulation of miRNA expression or activity. The majority of these studies have utilized in vitro culture systems to examine the effects of dietary constituents on miRNA expression. The few whole-animal studies that have been conducted have shown promise in their abilities to modulate miRNA expression [43]. This is the first report of dietary modulation of microRNAs in a spontaneous cancer model.

**Figure 8:**
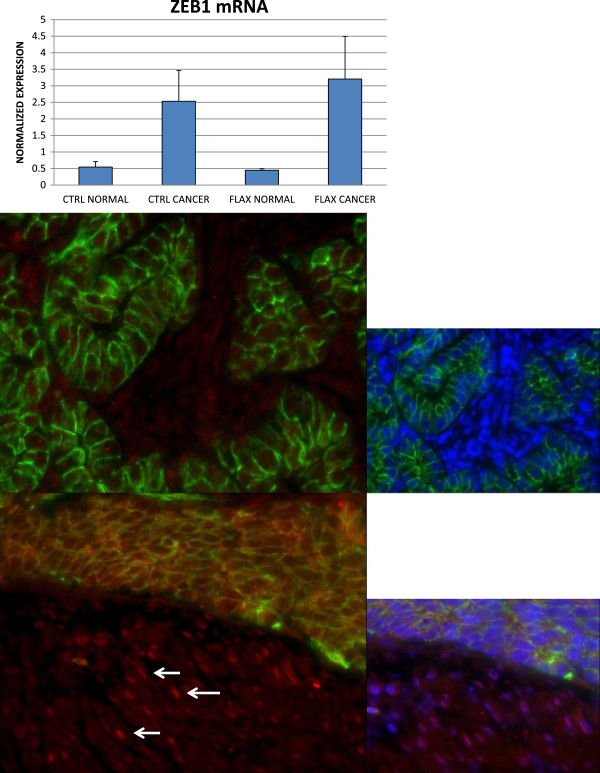
**ZEB1 mRNA and immunohistochemistry.** qPCR of *ZEB1* mRNA indicates an upregulation in tumors from both control and flax fed hens but no nuclear expression by immunohistochemistry in the tumor epithelium (control-fed top, flax-fed bottom). Some nuclear expression is seen in adjacent normal stroma from flax fed hen (bottom, arrows). *ZEB1* is red, E-cadherin is green, nuclei are stained with DAPI. Smaller micrographs include the blue DAPI channel. Magnification at 100x.

It has been well-established that the level of E-cadherin expression can reflect a balance between miR200 and ZEB1 expression [44]. The 3′ untranslated region of ZEB1 mRNA has several binding sites for the miR200 family and has been shown to be targeted for degradation by miR200. In turn, ZEB1 acts to repress transcription of both miR200 and E-cadherin [45–47]. We considered the possibility that the decrease observed in both E-cadherin and miR200 expression in tumors from flax-fed hens reflected increased ZEB1 expression. Analysis of *ZEB1* mRNA by qPCR indicated that there was already a significant increase in *ZEB1* mRNA in ovarian tumors from control-fed hens compared to normal, and a similar increase was observed in tumors from flax-fed hens compared to normal ovaries (Figure 8). Interaction between microRNAs and their mRNA targets can be mediated by degradation of the mRNA or by sequestration from translation. Immunohistochemistry was performed to determine if ZEB1 was expressed in the tumor epithelium. Oviduct served as a positive control and no staining was observed in normal ovary (Additional file 3). Immunohistochemistry revealed that there were ZEB1 positive cells in the normal stromal compartment (Figure 8, arrows) adjacent to the tumors but not in the tumor epithelial cells, nor in the stromal cells between the glandular tumor epithelia. No difference in localization was observed between tumors from control-fed and flax-fed hens. This suggests that the effect of the flaxseed diet on E-cadherin and miR200 in ovarian tumors in the chicken is not by way of an epithelial-mesenchymal transition mechanism caused by an increase in ZEB1 expression in the epithelial compartment.

Dietary flaxseed significantly decreases expression of miR-200 family in hen ovarian cancer, but does not affect expression of miR-200 family in normal ovaries. This observation is significant in its implication that some of the chemopreventive mechanisms of flaxseed function at the epigenetic regulatory level. Indeed, whole flaxseed contains elements that exert pleiotropic actions in cancer cells by functioning as antioxidant, anti-inflammatory, and anti-estrogenic agents.

## Conclusions

Microarray analysis revealed that flaxseed downregulates certain genes associated with ovarian cancer development and progression, and that pathways known to be dysregulated in ovarian cancer are targets of flaxseed action. More importantly, these data support the idea that dietary manipulation can modulate epigenetic and transcriptional changes associated with cancer development and progression. Notably, flax affects a group of genes in tumors that control branching morphogenesis during gland development, including *PAX2, FOXA2, MSX2* and *EN1*. Expression of these genes explains the glandular appearance of the tumors and is evidence of the process directing tumor growth, a process that involves proliferation and subsequent differentiation into glands. The upregulation of E-cadherin is a key feature of gland development and in these tumors is paralleled by the expression of miR-200 family members. Flaxseed downregulates all of these genes in a parallel fashion, suggesting a coordinated regulation, and without inducing an epithelial-mesenchymal transition; the epithelial morphology is maintained. Recently, stem-like epithelial cells have been identified in both ovarian surface epithelium and the distal cells of the tubal fimbriae [48, 49]. Induction of genes involved in glandular morphogenesis may drive these stem cells to proliferate and differentiate into the ovarian cortex in response to morphogens present in the cortex. We suggest that flaxseed reduces, but may not completely eliminate, signals from the cortex that are involved in the proliferative phase of the process of branching morphogenesis, leaving the ability to differentiate intact, thereby revealing molecular targets that will provide the foundation for clinical intervention studies.

## Methods

### Reagents

Antibodies: E-cadherin (BD transduction laboratories), PAX2 (Invitrogen), Dylight-488 donkey anti-mouse IgG, Dylight-549 donkey anti-rabbit IgG and Alexafluor-549 donkey anti-mouse IgG (Jackson Immunoresearch), DAPI fluorescent mounting medium (Southern Biotech). The HNF3B (HC7) monoclonal antibody was developed by Thomas Jessel and Susan Brenner-Morton and the Engrailed-1 monoclonal antibody (4D9) was developed by Corey Goodman. Both were obtained from the Developmental Studies Hybridoma Bank developed under the auspices of the NICHD and maintained by the University of Iowa, Dept. of Biology, Iowa City, IA 52242.

### Animal care and tissue collection

Single–comb White Leghorn hens were maintained as previously described [14, 50], with review and approval of the Institutional Animal Care and Use Committees at the University of Illinois at Urbana-Champaign and Southern Illinois University at Carbondale. The one year study included 387 2.5 year old hens and the five year study included 682 hens that aged 12–45 months. Hens were randomly divided into Control and Flax groups, where Control hens were fed a standard diet, while the Flax group was fed a diet supplemented with 10% flaxseed. Diet composition was previously described in detail [14]. Upon necropsy, tissues were collected and processed as described [14].

### Total RNA extraction and analysis

Total RNA was extracted from ovarian tissue that was either flash-frozen in liquid nitrogen, or stored in RNAlater (Invitrogen Life Technologies, Gaithersburg, MD). TRIzol reagent (Invitrogen) was used according to manufacturer′s instructions. Quantification of RNA was done using NanoDrop ND-1000 spectrophotometer measurement (NanoDrop Technologies). Integrity of total RNA was confirmed by Experion RNA StdSens Analysis (BioRad, Inc.). Biological replicates used in the microarray analysis were: 6 control normal replicates (C7,C8, C13, C22, C23, C31), 6 control cancer replicates (C16, C21, C30, C35, C60, C71), 6 flaxseed normal replicates (F1, F2, F5, F9, F20, F32) and 6 flaxseed cancer replicates (F11, F32, F36, F38, F86, F89) from the one year flaxseed study. The cancer replicates were of similar grade, stage and histotype.

### Labeling and hybridization

The microarray procedure was conducted at the University of Illinois Urbana-Champaign at the Keck Center for Biotechnology. One microgram of total RNA was labeled using the Agilent two-color QuickAmp labeling kit (Agilent Technologies) according to the manufacturer’s protocol. Agilent custom 4x44K chicken long oligo microarray, designed by Dr. Zhou of Texas A&M University was utilized for the array analysis [16]. Samples were hybridized using the *In situ* hybridization kit plus (Agilent Technologies, Palo Alto, CA, USA). Arrays were incubated at 65°C for 17 hours in Agilent’s microarray hybridization chambers. After hybridization, arrays were washed according to the Agilent protocol. Arrays were scanned at 5-μm resolution using an Axon GenePix 4000B scanner (Molecular Devices Corporation, Sunnyvale, CA) and images were saved as TIFF format. Images were quantified using Axon GenePix 6.0 (Molecular Devices Corporation, Downingtown, PA), and data were saved as .txt files for further analysis.

### Data normalization and statistical analysis

Median foreground signal intensities (no background subtraction) were normalized using Locally Weighted Linear Regression (LOWESS) within the R statistics package (version 2.7.2) using the “VSN” method in limma (version 2.14.7) to remove signal intensity-dependent dye bias. Spots with -100 flags were weighted zero before normalization. *P* value and fold changes between each comparison for each gene were calculated. Microarray data are MIAME compliant and available in Gene Expression Omnibus (GEO, http://www.ncbi.nlm.nih.gov/geo/) through the accession number GSE40376. Differentially expressed genes were identified by setting the significance level to a false discovery rate of <0.1.

### Bioinformatic analysis

Annotations were done using Database for Annotation, Visualization and Integrated Discovery (DAVID) tool [51, 52]. We applied various bioinformatics tools such as Multiple Experimental Viewer for the heatmap [53], and Ingenuity Pathway Analysis (Ingenuity® Systems, http://www.ingenuity.com). Functional classification of these genes was carried out using the gene expression analysis tool PANTHER (Protein ANalysis THrough Evolutionary Relationships) [54, 55] and Gene Ontology Enrichment Analysis Software Toolkit [56] for analysis.

### Microarray data sorting and gene expression analysis

Comparing the gene expression levels of the groups, the threshold level was set at >2 and <2 fold differences for the analysis. Differentially expressed genes in control-cancer vs. control-normal (CC-CN) and flax-cancer vs. flax-normal (FC-FN) constituted primary gene dataset. Then these primary datasets of CC-CN and FC-FN were compared which resulted in a secondary dataset consisting of 324 common and 287 uncommon genes of these two groups. The common genes signify that these genes are crucial in cancer progression and unaffected by flaxseed, whereas uncommon genes present in CC-CN group may be possible targets of flaxseed. To identify these potential flaxseed target genes, the uncommon genes in CC-CN were compared with flax-normal vs control-normal (FN-CN) dataset which generated a final list of 118 common genes.

### PCR-array

Four biological replicate samples from control-normal, control-cancer, flax-normal and flax-cancer were used for analysis. Total RNA was transcribed into cDNA using qScript DNA supermix. A customized 384 well StellARray (Cat#00194810) for *Gallus gallus* was purchased from Bar Harbor BioTechnology. A total of 44 target genes (including 4 housekeeping genes) were selected for analysis. Real-time PCR was performed using cDNA and EvaGeen mix (BioRad, Inc.) in a 384 well plate with primer mix for selected genes. The reaction and signal were measured using BioRad CFX manager software (BioRad Inc.). The expression levels were calculated as relative expression normalized to the expression levels of the housekeeping genes TATA box binding protein 1 (*TBP1*), Ribosomal protein L4 (*RPL4*), Glyceraldehyde 3-phosphate dehydrogenase (*GAPDH*) and Succinate dehydrogenase complex, subunit A (*SDHA*).

### cDNA Synthesis and qPCR analysis of mRNA Targets

First-strand cDNA synthesis was performed using total RNA and qScript cDNA Supermix (Quanta Biosciences 95048) according to manufacturer’s instructions. qPCR was performed using SsoFast EvaGreen Supermix (BioRad 172–5203). Reactions were 10ul and used 400 nm symmetric primer mix. Amplification conditions were as follows: 95C 5 s, 40 cycles for 95C 5 s, 58-68C 2 s. Expression analysis was performed using BioRad CFX Manager Software. mRNA levels were normalized to two stably expressed reference genes, *SDHA* and *RPL4*.

### Double-fluorescent Immunohistochemistry

Ovary tissue was collected from hens in the five year study, processed, fixed in NBF and paraffin-embedded as previously described [50]. Five micrometer sections were mounted onto charged Superfrost slides, deparaffined in xylene and rehydrated in graded ethanol solutions. Antigen retrieval was performed by heating slides under pressure of 15 psi in 0.1 M sodium citrate for 20 minutes. Slides were blocked with phosphate buffered saline/0.1% Tween-20 (PBST) and 5% fetal calf serum for 2 hours. Following blocking, sections stained for PAX2 and E-cadherin were incubated overnight with both rabbit anti-mouse PAX2 at 1:200 and mouse anti-human E-cadherin at 1:500 in PBST with 5% fetal calf serum. Slides were washed in PBST and incubated with both Dylight-488 donkey anti-mouse IgG and Dylight-549 donkey anti-rabbit IgG) at 1:200 for 2 hours. Sections stained for E-cadherin/EN-1 and E-cadherin/FOXA2 were incubated with anti- E-cadherin overnight, washed in PBST and then incubated with Alexafluor-549 donkey anti-mouse IgG at 1:200 for 2 hours. A second overnight incubation with either anti-HNF3beta or anti-Engrailed-1 at 1:10 in PBST and 5% fetal calf serum, followed by incubation with Dylight 488-conjugated donkey anti-mouse IgG at 1:200 for 2 hours completed the double-labeling. All slides were mounted with DAPI fluorescent mounting medium and visualized by confocal microscopy using a Leica model DM5500Q microscope using filters A4, Y5,and L5, and images were captured with a Leica DFC365 FX camera. Dual images were produced using Leica Application Suite-Advanced fluorescence version 2.6.0.7266.

### miRNA quantification

Total RNA was isolated from flash-frozen ovary tissue from the five year study using Tri Reagent (Ambion). First-strand cDNA synthesis was performed using Universal cDNA synthesis kit (Exiqon 203301). miRNA expression was quantified using SYBR Green master mix, Universal RT kit (Exiqon 203420). Locked nucleic acid primers for miR-200a (Exiqon 204707), miR-200b (custom Exiqon primer set), and miR-429 (Exiqon 205068) were used for quantification. qPCR values were normalized to two reference miRNAs stably expressed across the sample population, miR-460 (Exiqon) and miR-455 (Exiqon). Six samples were analyzed per group. Statistical analysis using one-way ANOVA followed by Student–Newman-Keuls post test was performed using GraphPad Instat program. p values of 0.05 or less were considered significant.

### miRNA *in situ*hybridization

In situ hybridization for miR-200a was performed as previously described [57] with modifications. Briefly, formalin-fixed, paraffin-embedded tissues from the five year study were sectioned at 5 micrometers and mounted onto positively-charged Superfrost slides. Sections were deparaffined in Histoclear and rehydrated through graded ethanol solutions. Sections were then digested with proteinase K (20ug/ml for 15 minutes) and acetylated. miR-200a double-DIG-labeled LNA probe (Exiqon) was diluted to 20 nM in hybridization buffer (Roche) and sections were hybridized overnight at 54°C. Following stringency washes, sections were blocked and incubated with goat anti-digoxin antibody conjugated to AP at 1:500 for 16 hours. Color development was performed using BCIP/NBT (Roche) in NTMT, 10% PVA and Levamisole (Sigma) for 30 hours. Sections were counterstained with nuclear fast red and mounted for visualization using a Leica model DM IL microscope and DFC 400 camera.

### Availability of supporting data

Microarray data are MIAME compliant and available in Gene Expression Omnibus (GEO, http://www.ncbi.nlm.nih.gov/geo/) through the accession number GSE40376.

## Electronic supplementary material

Additional file 1: **Relative expression of 337 genes related to the pathogenesis of ovarian cancer in pair-wise comparisons.** ^a^CCvsCN—control cancer vs control normal. ^b^FCvsCN—flax cancer vs control normal. ^c^FCvsCC--flax cancer vs control cancer. ^d^FCvsFN—flax cancer vs flax normal. ^e^FNvsCC—flax normal vs control cancer. ^f^FNvsCN—flax normal vs control normal. (XLS 70 KB)

Additional file 2: **118 flaxseed-responsive genes involved in ovarian cancer.** ^a^CC-CN-- control-cancer vs. control-normal. ^b^FC-FN--flax-cancer vs. flax-normal.^c^ FN-CN--flax-normal vs control-normal. (XLS 31 KB)

Additional file 3: **ZEB1 expression in oviduct and ovary, Top, expression of E-cadherin (green) in epithelium and ZEB1 (red) in adjacent stromal compartment of oviduct.** Middle, expression of smooth muscle actin (green) and ZEB1 (red) in the stromal compartment of the oviduct. Bottom, E-cadherin expression in the ovarian surface epithelium (green), no specific staining for ZEB1 (red) in normal ovary. Nuclei are stained with DAPI. (PDF 210 KB)
