# Supplementary figures and images for: Uncovering molecular events associated with the chemosuppressive effects of flaxseed: a microarray analysis of the laying hen model of ovarian cancer
Source: BMC Genomics. 2014 Aug 24;15(1):709. doi: 10.1186/1471-2164-15-709 (PMC4158050; doi:10.1186/1471-2164-15-709)

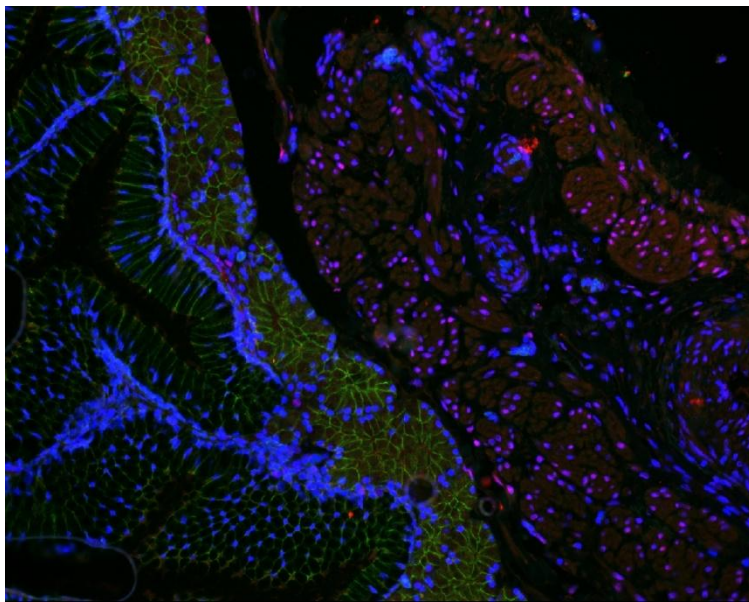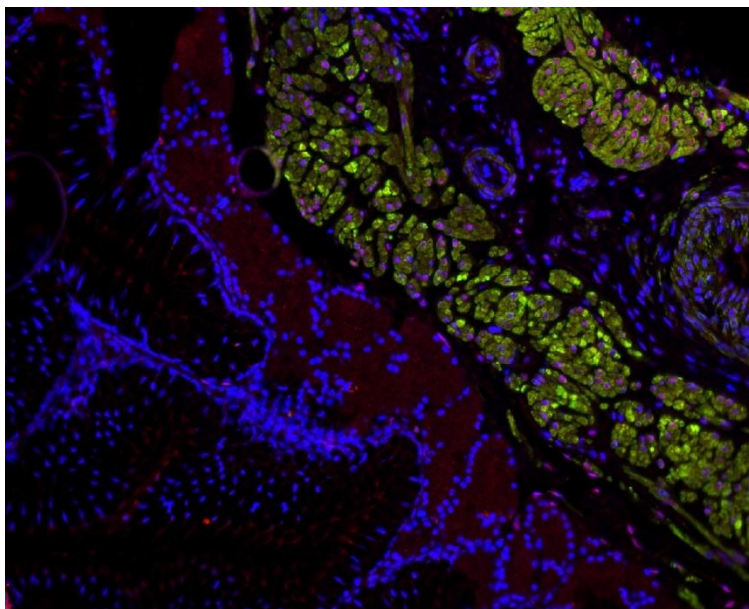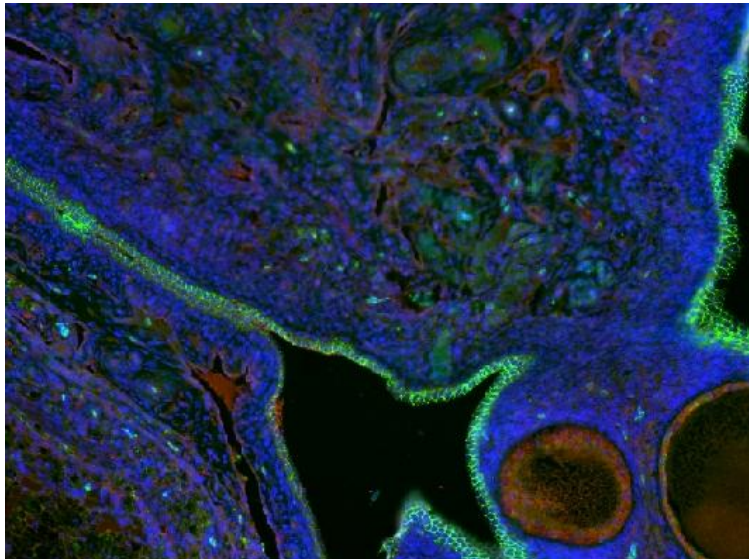

Supplement: Supplementary file 3 — Additional file 3: ZEB1 expression in oviduct and ovary, Top, expression of E-cadherin (green) in epithelium and ZEB1 (red) in adjacent stromal compartment of oviduct. Middle, expression of smooth muscle actin (green) and ZEB1 (red) in the stromal compartment of the oviduct. Bottom, E-cadherin expression in the ovarian surface epithelium (green), no specific staining for ZEB1 (red) in normal ovary. Nuclei are stained with DAPI. (PDF 210 KB) [file 12864_2013_6405_MOESM3_ESM.pdf]
